# Biochar from Biosolids Pyrolysis: A Review

**DOI:** 10.3390/ijerph15050956

**Published:** 2018-05-10

**Authors:** Jorge Paz-Ferreiro, Aurora Nieto, Ana Méndez, Matthew Peter James Askeland, Gabriel Gascó

**Affiliations:** 1 School of Engineering, RMIT University, GPO Box 2476, Melbourne 3001, Australia; s3232940@student.rmit.edu.au; 2 Departamento de Producción Agraria, E.T.S.I. Agronómica, Alimentaria y de Biosistemas, Universidad Politécnica de Madrid, Ciudad Universitaria, 28004 Madrid, Spain; gi.vare@upm.es (A.N.); gabriel.gasco@upm.es (G.G.); 3 Departamento de Ingeniería Geológica y Minera, E.T.S.I. Minas y Energía, Universidad Politécnica de Madrid, C/Ríos Rosas No. 21, 28003 Madrid, Spain; Anamaria.mendez@upm.es

**Keywords:** biochar, biosolids, soil amelioration

## Abstract

Ever increasing volumes of biosolids (treated sewage sludge) are being produced by municipal wastewater facilities. This is a consequence of the continued expansion of urban areas, which in turn require the commissioning of new treatment plants or upgrades to existing facilities. Biosolids contain nutrients and energy which can be used in agriculture or waste-to-energy processes. Biosolids have been disposed of in landfills, but there is an increasing pressure from regulators to phase out landfilling. This article performs a critical review on options for the management of biosolids with a focus on pyrolysis and the application of the solid fraction of pyrolysis (biochar) into soil.

## 1. Introduction

### 1.1. Biosolids Characteristics, Composition and Use in Agriculture

Sewage sludge is the solid fraction separated during wastewater treatment, while biosolids are treated sewage sludge, with the aim of meeting local heavy metal and pathogen regulations for possible land disposal. These terms are often confounded in the literature, for the purpose of this review, the authors employed the word ‘biosolids’.

The progressive implementation of the Urban Waste Water Treatment Directive 91/271/EEC [1] in European Member States is increasing the amounts of biosolids requiring disposal.

The Sewage Sludge Directive 86/278/EEC [2] is aimed at promoting the use of biosolids, while preventing undesired effects on soil, vegetation, animals and humankind. To achieve this aim, it prohibits the utilisation of untreated sludge on agricultural land unless injected or incorporated into the soil. Treated sludge (biosolids in the context of this article) is defined as having undergone “biological, chemical or heat treatment, long-term storage or any other appropriate process so as significantly to reduce its fermentability and the health hazards resulting from its use” [2].

Once treated, sludge can be recycled or disposed of using three main routes: reuse (land application), incineration or landfilling.

Five countries in the EU (France, Germany, Italy, Spain and the UK) produced 72% of the biosolids, where land application was the main final utilization. .Reuse (including land application and composting) seemed to be the predominant choice for management in EU-15 (53% of produced product), followed by incineration (21% of the product) [3]. This contrasted with practices in the twelve countries which joined the EU after 2004, where the predominant use was landfilling.

### 1.2. Properties of Biosolids and Land Application

The physico-chemical properties of biosolids depend on treatment technology and retention time in the wastewater facilities [4]. Stabilization technologies include composting, heat treatment, the addition of lime and anerobic digestion. Typical chemical composition and properties of untreated sludge are reported in Table 1. The chemical composition of the biosolids produced at a particular plant varies with time and season. Biosolids, following the stage of mechanical dewatering are masses presenting a lumpy structure and a bulk density within the range from 650 to 800 kg m^−3^ [4]. The dry solid content of biosolids oscillates 2–12% by weight [5] and is one of their most important parameters, when waste-to-energy management is considered.

Biosolids are rich in organic matter (usually more than 50% of the dry matter), mostly hydrocarbons, amino-acids or lipids and with a small presence of lignin or cellulose [9]. Its content in urban biosolids elevated but varies according to the treatment and conditioning carried out on the biosolids. Organic matter content may be reduced, as a consequence of dilution, for example following lime incorporation. The organic matter present in biosolids can lead to an amelioration of soil physical properties, including an improvement in soil structure or attenuating the potential for surface runoff and erosion. The mineralisation of the organic matter releases macro and micronutrients essential for crop development, reducing mineral fertiliser use. Organic matter contributes as an energy source for micro-organisms inhabiting in soil. Therefore, biosolids application may increase soil microbial population and activity [10].

Much high profile research has been performed in the recent years to obtain profit from waste, including the conversion to chemical and the valorization of nutrients [11]. Biosolids contain valuable nutrients (mostly nitrogen and phosphorous) which are available after land disposal but also possess a range of pollutants. In untreated sludge nitrogen contents typically range from 3 to 6% d.w. [4], while phosphorous is within 0.8 to 3.1% [4,5,12]. The majority of the nitrogen in biosolids is in organic form, although amounts of ammonium are found [9]. Thickening and dewatering of the sludge would result in a loss of ammonium, as most is in the liquid phase. Storage conditions can reduce N content by 30% [2]. Nitrogen availability is partially controlled by the type of treatment and usually is in accordance with the following rank: Composted sludge < anaerobic digested sludge < aerobic digested sludge.

The agricultural value of biosolids is also dependent on the mineralization rate of the organic N pool. N availability in biosolids is also affected by extrinsic soil factors, including climate, soil pH, soil texture and microbial composition. Loss of nitrogen can take place via volatilisation of ammonia or nitrate leaching. Nitrate leaching is of particular concern when biosolids are used for the reclamation of degraded areas, as this requires higher application rates [13]. However, this has been the preferred utilization in some countries [14]. Fast degradation of organic matter in the biosolids or higher doses of biosolids amendment could also lead to groundwater pollution induced by nitrate leaching [9].

Mineral form is the usual predominant fraction of phosphorus in biosolids, representing 30 to 98% of the total phosphorus [1]. Phosphorus content deserves particular interest, as it is a non-renewable resource. As with nitrogen, the amount of available phosphorus in biosolids depends on the treatment in the wastewater and is not directly related to the total phosphorus. Obviously, the contents of phosphorus in biosolids are significantly larger when a specific tertiary wastewater treatment for phosphorus removal is performed. Composted biosolids have lower phosphorus content due to a dilution effect caused by the low phosphorus content of additive products used when composting biosolids. Phosphorus content in biosolids is maintained after storage [2]. Phosphorus is classified as both fundamental and critical raw material to Europe’s economy, withChina being the main global supplier with 38% of phosphate rock production, [15] for this reason European countries are looking for new P sources, with the extraction from biosolids being one of the most novel techniques [15,16].

Lime addition to biosolids is performed for stabilisation purposes [17]. Lime treated biosolids have positive impacts on soil pH, cation exchange capacity and nutrient content with a positive effect on crop production [18,19]. Lime treated biosolids are particularly useful in soils whose productivity is limited by calcium. Carbon sequestration benefits have also been considered on occasions [20], although there is considerable uncertainty over the timescale at which carbon sequestration can be achieved.

### 1.3. Heavy Metals, Organic Pollutants and Pathogens in Biosolids

Biosolids tend to accumulate heavy metals and poorly biodegradable trace organic compounds as a consequence of the physical-chemical processes involved in wastewater treatment as well as potentially pathogenic organisms [21]. Contents of heavy metals in biosolids are within a wide range, depending on domestic effluents, road runoff, and industry in the area, with typical concentrations reported in Table 2.

Biosolids-borne heavy metals could enter the food-chain via plant uptake from polluted soils. Thus, the determination of metals in biosolids is a requirement for land application and is strongly regulated in environmental legislation. For example, EC Directive 86/278/EEC in Europe regulates the use of biosolids in soil and is built upon the heavy metal contents of both biosolids and sludge. Much less information is available concerning the presence and environmental fate of nanoparticles in biosolids. For example, silver nanoparticles can be released from clothing and have been detected in biosolids in non-labile forms [22]. Much work remains to be done to understand the fate and toxicology of nanoparticles in the environment.

A range of organic chemicals, including polycyclic aromatic hydrocarbons (PAH), polychlorinated biphenyls (PCBs), polychlorinated dibenzo-*p*-dioxins (dioxins, PCDDs), polychlorinated dibenzofurans (furans, PCDFs), perfluoroalkyl substances (PFAS) [23] may be found in biosolids. They remain unaltered after wastewater management (mesophilic, thermophilic or anaerobic digestion). During the 1980s and 1990s, the determination of pollutants in biosolids was focused on inorganic contaminants, pathogenic organisms, PAH, PCBs and PCDD/Fs (see for example, [23]).

Concerning PCDD/F, data have been taken from the *Compilation of EU Dioxin exposure and health data* [24]. It shows that average concentrations of dioxins are within the range of 15 and 40 ng I-TEQ/kg DM for EU countries. This would indicate that the sources of contamination in the Member States are similar. In some cases, concentrations over 1000 ng I-TEQ/kg DM have been reported.

Outside the scientific literature, there are no data from the Member States concerning most organic compounds. The EU has been debating for a decade and a half regulations for organic compounds in biosolids, but as yet, there are no guidelines for organic contaminants. As a consequence, no surveys have been regularly performed characterizing the organic pollutant content in biosolids. Some studies, show however areas for concern. If Danish regulations were implemented at the EU level, British biosolids would exceed the maximum allowable concentration of PAH by almost one order of magnitude [25]. Later work [26] has demonstrated that PAH content in biosolids would be the main limiting factor for its use as an agricultural product if more restrictive regulations were implemented.

A report from the EU has evaluated 114 of these chemicals in biosolids samples from 15 countries [27]. Only 8 countries in the EU (Austria, Belgium, Denmark, France, Germany, Sweden, Czech Republic and Slovenia) have regulations concerning the maximum concentrations allowed for organic pollutants in biosolids. An in-depth review on the regulation, risk assessment and types of organic pollutants in biosolids has been performed by Clarke and Cummings [26] and is outside the scope of this article. If biosolids quality does not improve, a large percentage of it would not be fit for land application in the future, as the EU is reviewing current regulations on persistent organic pollutants in biosolids. However, co-composting biosolids with other waste streams would allow to produce a material with a higher value and which can be applied to the land under these more restrictive regulations. Other organic pollutants in biosolids, which have been more recently the focus of attention include antimicrobials, pharmaceuticals, personal care products, surfactants and hormones. While individually, most of these products would not accumulate in the food chain at concentrations which would pose a risk for human health, the sum of them could be of concern [28].

Biosolids contain several type of micro-organisms, including a limited number of human and plant pathogens. Pathogens in biosolids include viruses, bacteria, protozoa, and helminths. Pathogens may be found in biosolids under infectious form but also as free living or resting forms (spores for bacteria or cysts and eggs for parasites) which will become pathogenic when they reach the human receptor. Their load in biosolids varies with time. Within the EU 11 out of 27 countries have limits for the content of pathogens in biosolids. In most cases, these limits concern helminth eggs, enterovirus and salmonella.

## 2. Management Alternatives for Biosolids

### 2.1. Incineration

The incineration of biosolids, with or without energy recovery, has a number of advantages benefits. There are multiple hearth and fluidized bed technologies available for biosolids incineration, the last one being the most prevalent [29]. Incineration reduces biosolids volume by about 70% and leads to the thermal breakdown of pathogens and toxic organic compounds [5,30]. Biosolids composition and heating value are the main input data for the evaluation of heat balances in biosolids incineration. The lower heating value of sludge is over the range 13.1–17.0 MJ/kg [5,9], similar to that of brown coal.

However, incineration is a costly alternative due to the external requirements of energy associated with dewatering. Incineration must comply with regulations in air pollution. In the case of EU the relevant regulation is Directive 2000/76/EEC on waste incineration. New technologies have allowed the maintenance of gaseous emissions within regulatory levels, but have also contributed to an increase in the costs of incineration. Co-incineration with municipal solid waste is becoming increasingly popular compared to mono-incineration. However, the ash originated from the co-combustion of coal and biosolids is potentially more toxic than the ash from coal, due to the large amounts of heavy metals [31]. Biosolids incineration is perceived poorly by the community, and, as a consequence, other methods of management are preferred.

### 2.2. Landfilling

Landfilling has been a common disposal practice for biosolids, due to reduced costs, but it is becoming increasingly restricted in different countries. Following Directive 75/442/EEC and Directive 91/156/EEC the least desirable option for biosolids disposal is landfilling. The Landfill Directive 99/31/EEC [1] aimed to reduce the quantity of biodegradable municipal waste to 35% of that landfilled in 1995 by 2016. In addition, the cost of the land needed for landfill is increasing in Europe and elsewhere as a consequence of competing land uses. The high amount of organic matter present in the biosolids contributes to methane emissions from landfills and to leachate. Thus, landfilling is facing also an increased public opposition over these environmental concerns.

### 2.3. Pyrolysis

Pyrolysis can be a potential promising method for biosolids management as, compared to other alternatives uses, pyrolysis has a lower carbon footprint [32]. Pyrolysis offers the advantage of delivering less flue gas to be managed as compared to incineration. Simultaneously, it diminishes the amount of acidic gases and dioxins formed.

Pyrolysis can broadly be categorized as slow pyrolysis and fast pyrolysis. Slow pyrolysis, operates at long residence times and slow heating rates, maximizing the solid fraction (biochar or activated carbon) at the expense of the energy product (bio-oil or py-gas). Fast pyrolysis undertakes the thermo-chemical conversion at a rapid heating rate (around 100 °C/min), which maximizes the liquid and gaseous fractions. Pyrolysis can produce a higher yield of py-gas and higher hydrogen content from the conversion of wet biosolids than from dry ones [33].

Increasing the temperature of pyrolysis maximizes the yield of the gaseous fraction and decreases the solid fraction, while the liquid fraction remains constant [34]. Heating rate is an important parameter, but only for temperatures below 650 °C [34]. Thus, with increasing heating rate the composition of the pyrolysis oils is shifted towards lighter compounds, as a consequence of cracking conditions being favored [34].

Traditionally, several avenues have been explored for the pyrolysis of biosolids. This includes the valorization of products through liquid production [35]. The liquid obtained is heterogeneous, with fractions having dissimilar calorific value [35], which complicates energy recovery. In addition, the bio-oil can be corrosive [36]. Higher energy recovery is possible when combining aerobic digestion with pyrolysis [37] or with gasification [38].

Biosolids pyrolysis has also been researched with the aim to acquire a solid to use as adsorbent [39,40,41], which could be used for example to improve the performance of an activated sludge reactor [41] or as a catalyst [42]. The specific surface area of chars produced from biosolids, after activated, can attain values around 360 m^2^ g^−1^, which is lower than for commercial activated carbons (500–1200 m^2^ g^−1^). However, the adsorbents obtained from biosolids pyrolysis may be more effective than activated carbon in order to eliminate contaminants such as H_2_S and NOx generated in thermochemical processes [43,44,45]. These chars have also found application to sorb metals, phenols and dyes [35].

Another traditional avenue for research was the possibility to use pyrolysis prior to landfill. Thus, pyrolysis reduces the potential release of pollutants from the resulting char in landfill, compared to the biosolids or an incinerated residue [46]. Thus, pyrolysis is a promising method of treating biosolids before landfilling, in order to reduce the leaching of pollutants and safe significant amount of space in landfills.

The approaches for valorization above have been reviewed multiple times. Valorization of biosolids and other types of waste, comprising approaches such as platform chemicals [11] or catalysts [11,42] have been reviewed. Much work has been devoted to review energy aspects of the pyrolysis of biosolids, with an emphasis on the gaseous and liquid fractions. This work has been carried out at a local [14] or international level [35,37]. However, to our knowledge, there is no review emphasizing the solid fraction of biosolids biochar and discussing its value as a soil amendment.

In recent years, however, there have been an increasing number of studies concerning the use of biochar as soil amendment due to its numerous advantages, including the improvement of the soil productivity [47], remediation of contaminated soils [48] and climate change mitigation [49].

In sum, as with incineration, pyrolysis reduces the volume of biosolids, eliminates pathogens. The resulting product from pyrolysis, biosolids derived biochar possesses high amounts of carbon and nutrients, and a significant cation exchange capacity. Biochar has a highly porous structure and significant amounts of extractable humic and fulvic acids [50]. Most of the carbon in the biochar can be highly recalcitrant, with carbon turnover on a timescale of millennia [51]. This product has demonstrated significant benefits to remove contaminants from wastewater [45] or to improve soil properties [47]. Biochar use into soil was initially spurred by its interest as a mean to sequester carbon [49] and reduce greenhouse gas emissions from soils or from waste management practices. However, an upsurging interest is the use of biochars as a way to immobilize organic or inorganic pollutants [52,53,54,55,56]. Biochar´s properties upon addition to soil will depend on the material used to produce it (feedstock), the type of pyrolysis reactor and the temperatures achieved during pyrolysis. All these are going to be discussed in detail in the following sections.

## 3. Biochar from Biosolids

### 3.1. Biochar Properties according to Preparation Method

Very little information is available concerning the influence of the type of pyrolysis unit on the final characteristics of biosolids biochar. The characteristics of biochar produced in traditional retort kilns and in muffle furnaces differ [57]. Thus, the biosolids biochar produced in the muffle furnace contained 70% more ash, 78% more fixed carbon, and 63% less volatile matter than the biochar produced in a traditional retort kiln. The traditional retort kiln biochar inhibited seed germination in Petri dishes and corn yield in a plot experiment, while opposite effects were observed when the muffle furnace biochar was utilized. Differences were attributed partly to the dissimilar amount of available N present in both biochars and to the presence of pollutants in the volatile matter of the traditional retort kiln biochar.

### 3.2. Biochar Properties Using Pyrolysis under N_2_ or CO_2_ Atmospheres

The dye adsorption capacity of biosolids biochar prepared from pyrolysis has been studied [40], under both N_2_ and CO_2_ atmospheric, in a temperature range of 350–750 °C. The results showed that, at higher temperatures, for chars prepared under both atmospheres, the porosity of the chars increased as a consequence of the removal of volatile matters during pyrolysis [58]. Similarly, BET surface area increased with temperature. The FT-IR spectra of the biochars prepared under both N_2_ and CO_2_ atmospheres indicated a decrease in –OH, –NH and –C=O functional groups at higher temperatures, which lead to an increase in the alkalinity of the char.

The basicity of both chars, on the other hand, increased with increasing pyrolysis temperature. This temperature effect is more pronounced at temperatures above 550 °C. At the same temperature, the surface basicity of the char prepared under the CO_2_ atmosphere was significantly higher than the one prepared under N_2_ atmosphere. The dye adsorption capacities of biosolids derived chars obtained from CO_2_ pyrolysis are higher than those obtained from N_2_ pyrolysis; moreover, biosolids derived char prepared from the pyrolysis/gasification (CO_2_-pyrolysis) possess higher surface area than that of prepared by pyrolysis under N_2_ atmosphere [40].

### 3.3. Biochar Physico-Chemical Properties according to Pyrolysis Temperature

Some of the seminal studies concerning the pyrolysis of biosolids with the intention to prepare a biochar for agronomic use are the ones by Hossain et al. [59,60]. According to Hossain et al. [60], pyrolysis temperature had a profound impact on the chemical properties of the biochar (Table 3). The results also showed that by increasing the pyrolysis temperature (within the range from 300 to 700 °C) the yield of biochar decreased. Similarly, later studies [21] also concluded that pyrolysis of biosolids at 300 °C maximized biochar yield. Molar rations H/C, O/C and N/C are reduced when biochar is produced at higher temperatures, indicating an aromatization of its structure [61].

In the study by Hossain et al. [60], biochar produced at a low temperature (300–400 °C) was acidic while the solid product shifted to an alkaline pH at high temperature (700 °C). This work demonstrated that by manipulating the temperature of pyrolysis it is possible to create biochars targeted for application in acidic or in alkaline soils. Other properties also showed stark variation with temperature. Thus, electrical conductivity increased slowly with temperature of up to 500 °C but halved at higher temperatures. This result is of utmost importance, as the salts borne by biosolids can be harmful for seed germination and plant productivity, when they accumulate in the rooting zone, particularly in arid areas, where leaching of salts is limited. Reclaimed lands, where biosolids can be applied at relatively high rates, would also be areas of concern.

N concentration in the biochar, particularly inorganic nitrogen, was relatively low and tended to decrease with temperature [60]. The total concentration of the main micronutrients (Ca, Fe, Mg, S, Cu and Zn) increased with temperature. The total P content in the biosolids biochar increased by 43% when pyrolysed at a temperature of 700 °C. A similar trend was reported for potassium. This suggested that phosphorus and potassium were associated with the inorganic fraction of the biosolids. Similarly, Roberts et al. [62] pyrolyzed biosolids with and without alum and found that, after pyrolysis, over 90% of the initial P content in the biosolids was present as plant-available phosphorus in the biochars. The effect on plant available phosphorus was more marked for biosolids containing alum.

Méndez et al. [63] studied the agronomic and physicochemical properties of biosolids biochar produced at 400 and 600 °C and determined that the volatile matter content of biochar decreased at higher pyrolysis temperatures, while the fixed carbon content was comparable. BET surface area, pH, porosity and total concentration of Cu, Ni, Zn, Cd and Pb in biosolids biochar increased with temperature, whereas and electrical conductivity and cation exchange capacity of biochar were lower than in the original biosolids and were further reduced at higher temperatures. This variation on biochar properties had an impact after soil amendment. Notably, soil available water and field capacity increased in soils amended with the biochar produced at 600 °C, while values for the control and the soil amended with the biochar produced at the lower temperature remained identical.

Like in previous works, a large impact on biosolids surface area was obtained by Antunes et al. [64]. However, in contrast with other researchers [62], their biochars had a similar pH to the original biosolids, differences which could be attributed to the type of pyrolysis. Thus, most of the work outlined in this review concerns biochars prepared from slow pyrolysis, while Antunes et al. [63] utilized a microwave pyrolysis technique.

### 3.4. Effect of Biochar from Sewage Sludge on Plant Yield and Soil Nutrients

Table 4 showcases the main studies concerning changes in soil fertility in soils amended with biosolids biochar. The use of biochar in soils can contribute to an increase in soil pH and cation exchange capacity [48], which would have generally benefits on plant yield and germination [65]. On the other hand, under certain conditions, some biochars can bind nutrients present in soil, limiting their accessibility for plants [65]. Bridle and Pritchard [66] ascertained that biochar produced from biosolids had low amounts of plant available N in spite of a low C:N ratio value of 7 and an elevated amount of total N (6.4%). This study reported that 45% of the initial nitrogen was not present in the final char, whereas losses of P during pyrolysis were negligible. In spite of this lower amounts of N present in comparison to the original biosolids, which has been corroborated by other studies [67], biosolids biochar contributes to retain soil nitrogen in the long-term, acting as a slow release fertilizer. Ammonium and nitrate leaching is thus reduced following biochar amendment [67].

Hossain et al. [59] used a biochar prepared in a fixed bed reactor at 550 °C. They found that the treatment with biochar resulted in a 64% increase of cherry tomato yield when compared to the control. Cherry tomato yield with the biochar treatment was lower than when an optimal amount of NPK fertiliser was applied to the soil. The highest yield (20% higher than in the fertilizer only treatment) was achieved by a treatment combining biochar and fertilizer application. This increase in plant productivity would have been mediated by increases in phosphorus and nitrogen in the treated soils. The authors concluded that biochar could partially offset, but not fully substitute, the use of inorganic fertilizers in cherry tomato. Paz-Ferreiro et al. [68] found a biochar produced from biosolids to be effective in increasing the number of fruit per plant and the plant biomass in both a Ferralsol and an Acrisol planted with millet. The results were even more noticeable when the earthworm Pontoscolex corethrurus was present in the pots. 

Wang et al. [69] prepared a biochar from a mixture of biosolids and eucalyptus. The wood material was intended to improve the calorific value of biosolids. Their study demonstrated that the P present in the feedstock was fully present in the biochars (prepared at temperatures from 250 to 550 °C). The biochars prepared increased grass yield in a pot experiment to the same extent than traditional P fertilizers. Overall, the biochars acted as a slow-release fertilizer. A later study of the same authors [70] studied the different forms of N in the same biochars. This second study concluded that, out of several N pools present in the biochars, total hydrolysable N, determined by acid hydrolysis with 6 M HCl correlated to the labile N in the biochar, while dichromate oxidisable N, which includes heterocyclic N, would be a N source in the long-term. Other authors have confirmed that some N is lost by volatilization during pyrolysis, while the simpler organic nitrogen compounds in biosolids are transformed into heterocyclic aromatic forms of nitrogen [72].

The interaction between soil and biochar can be altered over long periods of time. Biochar will be oxidised, and acidic functional groups from biosolids will be released to the soil solution, leading to a drop in the pH value, which can increase the phytotoxicity and the heavy metal availability [73].

Seed germination tests have been previously used to predict the phytotoxicity or phytostimulative effect of biochars. For example, Gasco et al. [71] found a biosolids biochar to be phytostimulant for lentil and lettuce, while no effect was found for cress, cucumber and tomato. Overall, much research needs to be conducted into the effects of biochar on plant signalling pathways and gene expression in order to be able to predict plant response after biosolids biochar addition.

### 3.5. Effects on Soil Biological Properties

Biological properties have often been used as indicators of soil quality, due to their sensitivity to external changes. Biosolids biochar addition often results in the increase of soil enzymatic activities [68,74]. The results of biochar addition on soil microbial community are soil-specific. For example, Paz-Ferreiro et al. [75] used PLFA to measure bacterial, fungal and actinomicetes populations in soils with and without biochar addition. They found those populations to increase in an Acrisol after biochar addition, but to diminish in a Ferralsol. Importantly, a change towards more fungal-dominated microbial communities was observed in this work concomitant with an increase in carbon availability. Increased fungal to bacterial ratios have been associated with more sustainable agricultural systems, partly as a consequence of a more efficient crop nutrient uptake mediated by mycorrhizal fungi.

### 3.6. Effects on Organic Pollutants 

Pyrolysis also has the advantage to remove organic pollutants from the biochar. Some of the common pollutants constituents of biosolids (namely the antibacterial agents triclocarban and triclosan and the surfactant nonylphenol) could be almost completely removed (below detection limit) from the biochar after being pyrolyzed for 2.5 min at 500 °C [76]. Hoffman et al. [77] pyrolyzed biosolids at temperatures ranging from 100 to 500 °C and found that pyrolysis at 400 °C for one hour diminished the estrogenicity, measured in estradiol equivalents, of the samples by 95%. 

Zielinska and Oleszczuk [78] found that conversion to biochar reduced total PAH content from 8- to 25-fold, with respect to the original biosolids. The total content of PAH in the biochars depended on the origin of the biosolids. In general, PAH increased with the temperature of pyrolysis, within the range of temperatures tested (500 to 700 °C). Importantly, pyrolysis resulted in a stark decrease of 5- and 6-ring compounds in the PAH mixture. Naphthalene was higher in the biochars than in the biosolids, but the toxicity of this compound is lower than for other PAHs. Pyrolysis of biosolids also reduces available PAHs [79,80]. These authors added biosolids and biosolids biochar at a concentration of 2, 5 and 10% to a soil planted with lettuce [79] or tomatoes [80]. The concentrations of PAHs in tomatoes were lower when biochar, and not biosolids, were added to the soil. For lettuce, bioaccumulation was reduced by 56% to 67% with respect to the control soil. Similar results were found for cucumber where reductions of PAH in the plant were in the range of 44 to 57% for biosolids biochar amended soils [81].

### 3.7. Effect on Greenhouse Gas Emissions from Soils

There is very little information concerning greenhouse gas emissions in soils amended with biosolids biochar. Méndez et al. [63] found, in a laboratory incubation, that biochar reduced the CO_2_ emissions by 11 (for biochar prepared at 400 °C) to 32% (for biochar prepared at 600 °C). After 10 years, this would equal to a reduction of CO_2_ emissions between 301 and 932 kg CO_2_-C ha^−1^ with respect to land application of biosolids. Khan et al. [82] found that biosolids biochar reduced N_2_O emissions from a paddy soil by 96% or 98%, depending on the dose applied. In the same experiment, biochar addition resulted in the soil shifting from being a source to a sink of methane.

### 3.8. Heavy Metal Content and Mobility

The content of heavy metals in highly variable from one wastewater treatment plant to another, with large variations reported from one community to another over short distances [83], and this will affect the heavy metal content in the pyrolysis product. Seasonal variations are also likely to occur [84]. Pyrolysis temperature has an effect on enrichment of heavy metals (Zn, Pb, Ni and Cd) in the produced biochar [60,83,85], which is important consideration as the heavy metals can bioaccumulate when biochars are applied to the soil. Most of the Hg (81 to 99%) and As (30–96%) originally present in biosolids are volatilized [82]. Lesser amounts of Se are also found in the biochar, with respect to the feedstock [83]. Cd content in biochar can decrease with pyrolysis, if temperatures are high enough, as a consequence of volatilization [78]. The total content of heavy metals could restrict land application of the biochars, depending on the initial heavy metal content in the biosolids and on regional or national regulations. In political entities where land application is restricted, alternatives uses can be developed, including the generation of energy by incineration. The co-pyrolysis of biosolids biochar with other feedstock could lower the concentrations of heavy metals to levels compatible with legislation, but it has seldom be attempted. Co-pyrolysis of biosolids with rice straw or sawdust reduces the total amount of heavy metals in the biochar, without any noticeable effect on heavy metal mobility [86]. Alternatively, carbon materials, including activated carbon [87] or biochar, and acidic treatments [88] could be used to diminish the content of heavy metals pre-pyrolysis.

Some recent work suggests that, after pyrolysis, the mobility and health risk of biochar are overestimated under current regulations for the land application of biosolids. For example, Hossain et al. [59] measured sixteen trace elements in tomatoes grown with biosolids biochar and found that, in all treatments, all sixteen elements were below the maximum concentration allowed by the Australian Food standard. A reduction in heavy metals in grown with biosolids biochar with respect to plants grown with biosolids was also found for cucumber [80]. An experiment reports accumulation of heavy metals, in particular Ni, in garlic tissues of a soil amended with biosolids biochar [89]. However, this study used doses of biochar which seem not to be realistic from an agronomic point of view, with additions of biochar ranging from 15 to 50%. Overall, the results obtained in plant-growth experiments are consistent with the reduction in the available and plant-extractable fractions of several heavy metals (Ni, Zn, and Pb and to a lesser extent Cd and Cu) which are reported by other authors [85]. A leaching experiment confirmed that Cu, Ni and Zn are less likely to contaminate the groundwater in soils amended with biosolids than in those amended with the same dose of biosolids [85]. A range of extractants have been used to demonstrate that biosolids biochar, particularly when prepared at higher temperatures, decreases the leaching of heavy metal, including diethylene triaminepentaacetic acid (DTPA) [85] and toxicity characteristics leaching procedure (TCLP) [61,90]. Sequential extraction analysis of heavy metals have demonstrated a shift towards the more stable oxidizable and residual forms, in particular when pyrolysis temperature is above 500 °C [91]. An ecological risk index derived from BCR extractions indicated that there is a gradual decrease of the risks associated to heavy metals present in biosolids at higher temperatures. The values of this index reached a value of 674 for biosolids, 432 for biochars prepared at 400 °C and decreased gradually until a value of 112 for biochars prepared at 600 °C [91].

Another interest area for research is the modification of biochars in order to facilitate their retention of heavy metals. Agrafioti et al. [21] pyrolyzed biosolids without chemical reagents added and biosolids impregnated with chemical reagents (K_2_CO_3_ or H_3_PO_4_) with the aim to increase the surface area of the biochars. Biosolids impregnation with K_2_CO_3_ resulted in a 5 times higher the surface area, compared to the non-impregnated sample, at 500 °C. In contrast, H_3_PO_4_ lead to a lower values of surface area in the biochar. This could have been related to the confinement of orthophosphates in the char. Based on the TCLP tests, the non-impregnated biochar was sample was more efficient in impeding the release of heavy metals. 

### 3.9. Biochar Combination with Composting

Biochar could be used in conjunction with other waste management techniques for mitigating environmental impacts. A biosolids biochar was used as an additive during the composting and vermicomposting of biosolids and wood chips [92]. During vermicomposting, the number of cocoons increased by 213% compared to the control after 4 weeks. After 6 weeks, the average number of juveniles increased by 11-fold. It is also known that greenhouse gas emissions during co-composting can be mitigated by the addition of biosolids biochar [93]. This higher reproduction rate would facilitate the conversion of the feedstock to compost. Awasthi et al. [93] found CH_4_ and N_2_O emissions to be over 90% lower when biosolids biochar was added in the process of composting. No significant differences were found for carbon dioxide. Biochar addition halved N losses as ammonia, which is transformed into nitrate in the presence of biochar [94]. The mechanism behind these changes in microbial-related processes is possibly related to the effect of biochar on the structure and succession of bacteria diversity during co-composting [95] during the thermophilic phase. A higher temperature in compost piles with biochar [94] could account for part of the effect observed. 

Heavy metal availability, a factor hindering the use of compost prepared from biosolids can also be reduced as a consequence of co-composting with biochar. Thus, Liu et al. [94] found the levels of available Cu, Cr, As and Pb to be significantly lower when biochar was used for co-composting. Similarly, biochar mitigated the availability of Cd and Zn to earthworms during vermicomposting [93]. 

### 3.10. Use of Biochar as a Growing Media

Recent research has examined the role of biosolids biochar as a substitute for peat in growing media, with the potential to be used for horticulture or as a landfill cover. At present, peat is extracted for its use as a growing media, with a number of environmental problems associated to this activity. Horticultural use, and the concomitant abatement of greenhouse emissions associated to peat exploitation could be better perceived by the public compared to the agricultural use of biosolids biochar. Mendez et al. [96] studied peat substitution by biosolids biochar or biosolids. A 10% substitution (by volume) was used. The addition of biosolids biochar resulted in an increase in the N, P and K contents of the growing media. More importantly, lettuce biomass increased by 184–270% and the shoot length by 137–147% with respect to the control (peat alone). This was in spite of hydrophysical properties being similar in the control and the biochar treatments. Microbial biomass increased by 966% in the treatments with biochar. The authors also verified that the presence of trace element (with the exception of lead in one of the biochars prepared) in the plant tissue was lower than in the control. Kaudal et al. [97] prepared biochar from the co-pyrolysis biosolids and greenwaste, finding that the proportion of particles in the desirable range for growing media (0.25 to 2 mm) was ameliorated in biochar mixes, when compared to coir peat.

Hydrochars prepared from biosolids were also suitable to replace peat (up to 50%). Peat-hydrochar mixtures presented similar enzymatic activity than peat alone [98]. Hydrochar addition to peat increased the air space, water holding capacity and consequently, total porosity when compared to peat alone. Grass (Lolium perenne) growth was higher in the hydrochar or in the hydrochar-peat mixture than in peat alone.

## 4. Conclusions

Our literature review has shown that there is scope to manage biosolids in a more sustainable manner Pyrolysis of biosolids leads to several benefits, compared to the traditional landfilling, incineration or land application. This includes few gaseous emissions, the destruction of pathogens, the potential to recover energy and a solid product which can be used as a soil amendment. The nutrient content of biochars prepared from biosolids is high, in particular for phosphorus. Paradoxically, very limited work exists concerning the use of biosolids biochar as a product to improve agronomic performance. This could be due to concerns of toxicity via the food chain, which seems irrational given the demonstrated ability of biochar to immobilize pollutants, in particular heavy metals. The use of biochar for growing non-edible plants in horticulture, as a substitute for other growing media materials, could mitigate these concerns. Alternatively, biosolids could be blended pre-pyrolysis with a material containing lower amounts of heavy metals. To manage biosolids in a sustainable manner, there is a need for further understanding about the soil-biochar-plant interaction. In this aspect, to ensure a safe use of biochar prepared from biosolids, there is an imperative need to perform experiments aiming to assess the long-term stability of biosolids biochar in soil and the long-term fate of nutrients and pollutants. 

## Figures and Tables

**Table 1:** Maximum metal concentration allowed in soils treated with sewage sludge in different countries (mg/kg) [6,7,8].

Country	Cr	Ni	Cu	Zn	Cd	Pb	Hg
European Union	100−150	30−75	50−140	150−300	1−3	50−300	1−1.5
Germany	100	50	60	200	1.5	100	1
Denmark	30	15	30	100	0.5	40	0.5
Spain	100−150	30−112	50−210	150−450	1−3	50−300	1−1.5
Finland	200	60	100	150	0.5	60	0.2
France	150	50	100	300	2.0	100	1
Italy	150	50	100	300	3.0	100	-
Norway	100	30	50	150	1.0	50	1
UK	400	75	135	300	3.0	300	1
Sweden	30	15	40	100	0.5	40	0.5
Netherlands	100	35	36	140	0.8	85	0.3
United States of America	1500	210	750	1400	20	150	8

**Table 2:** Comparison of the limit values for heavy metal concentrations in biosolids for use in agriculture (mg kg^−1^ of dry matter) between European Union and United States of America [6,7].

Metal	European Union		United States of America	
	Maximum Permitted Concentration in Sludge (mg kg^−1^)	Maximum Annual Loading (kg ha^−1^ y^−1^)	Maximum Permitted Concentration in Sludge (mg kg^−1^)	Maximum Annual Loading (kg ha^−1^ y^−1^)
Cr	-	-	3000	150
Ni	300−400	3	420	21
Cu	1000−1750	12	4300	75
Zn	2500−4000	30	7500	140
Cd	20−40	0.15	85	39
Pb	750−1200	15	840	300
Hg	16−25	0.1	57	0.85

**Table 3:** Main findings with respect to the physico-chemical characteristics of biochars derived from biosolids.

Study	Temperatures	Main Findings
Hossain et al. [60]	350, 400, 500, 700 °C	Higher pyrolysis temperature leads to less char but to less plant-available heavy metals (as measured by DTPA). Strong contrast in pH depending on temperature.
Agrafioti et al. [21]	300, 400, 500 °C	Impregnation of sludge catalyzes pyrolysis. Higher yield at lower temperature.
Chen et al. [61]	500, 600, 700, 800, 900 °C	Biochars outperform commercial activated carbon for heavy metal sorption. This is related to aromatization and development of pore structure at higher temperatures.
Roberts et al. [62]	300, 450, 600, 750 °C	Most P in biosoilds available for plants after transformation to biochar.
Méndez et al. [63]	400, 600 °C	Total amount of heavy metals increased with temperature, but metals were less extractable.
Antunes et al. [64]	300, 400, 500, 600, 700, 800 °C	pH similar to original biosolids. Surface area quadrupled at higher temperatures.

**Table 4:** Main effects of biochar on soil nutrients and plant yields.

Study	Soil Type (Classification System)	Temperature of Pyrolysis and Plant Species	Main Findings
Yuan et al. [67]	Ultiso, Typic Plinthudult (USDA)	300, 400, 500, 600, 700 °C	Biochars prepared at high temperatures reduced the leaching of nutrients.
Hossain et al. [59]	Chromosol (Australian)	550 °C. Cherry tomato	Plant weight, number of fruits and fruit yield increased, particularly when additional fertiliser was provided.
Paz-Ferreiro et al. [68]	Acrisol and Ferralsol (FAO)	600 °C. Proso millet	Increased plant productivity and number of fruits. Increased soil microbial activity, in particular in the presence of earthworms.
Wang et al. [69]	Entisol, Typic Udipsamment (USDA)	250, 350, 450, 550 °C. Italian ryegrass	Biosolids biochar have similar P contents and availability to commercial fertilizers.
Wang et al. [70]	No soil addition performed	250, 350, 450, 550 °C	The role of different N pools from biochars on long-term and short-term N availability is ascertained.
Gascó et al. [71]	Haplic Cambisol (FAO)	600 °C. Lentil, lettuce, cress, cucumber and tomato	Phytostimulant for lentil and lettuce, but not for cress, cucumber and tomato.
